# An analysis of stimulation methods used in rehabilitation equipment for children with cerebral palsy

**DOI:** 10.3389/fneur.2024.1371332

**Published:** 2024-06-18

**Authors:** Cunxiao Guo, Yongdan Cun, Bo Xia, Suyu Chen, Can Zhang, Yiping Chen, Exian Shan, Pengyue Zhang, Xiantao Tai

**Affiliations:** ^1^Yunnan Key Laboratory of Integrated Traditional Chinese and Western Medicine for Chronic Disease in Prevention and Treatment, Key Laboratory of Acupuncture and Massage for Treatment of Encephalopathy, College of Acupuncture and Tuina Rehabilitation, Yunnan University of Chinese Medicine, Kunming, Yunnan, China; ^2^Yunnan College of Business Management, Kunming, China; ^3^Master of Science in Computer Science, Sofia University, Palo Alto, CA, United States

**Keywords:** cerebral palsy, artificial intelligence, rehabilitation equipment, stimulation mode, review

## Abstract

**Objective:**

This paper summarizes the research progress into stimulation methods used in rehabilitation equipment for pediatric cerebral palsy (CP) for the past 20 years from 2003 to 2023. We also provide ideas for innovative research and development of artificial intelligence-based rehabilitation equipment.

**Methods:**

Through a certain search strategy, Keywords are searched in the China National Knowledge Network Database (CNKI), the Wanfang Database knowledge service platform, the Chongqing VIP information service, PubMed, Web of Science, Cochrane, ScienceDirect, Medline, Embase, and IEEE database. A total of 3,049 relevant articles were retrieved, and 49 articles were included that mentioned research and development of rehabilitation equipment. We excluded articles that were not specific to children with CP, were duplicated or irrelevant literature, were missing data, the full article was not available, the article did not describe the method of stimulation used with the rehabilitation equipment on children with CP, were not Chinese and English, and were the types of reviews and commentaries.

**Results:**

Physical stimulation is the main stimulation method of rehabilitation equipment for children with CP. Force stimulation is the main mode of physical stimulation, and there are 17 articles that have verified the clinical efficacy of force stimulation-based equipment.

**Conclusion:**

Research on the stimulation mode of pediatric cerebral palsy rehabilitation equipment is likely to focus on simulating the force of the Chinese medicine called “tuina manipulation.” When this method is combined with artificial intelligence and personalized direction we believe this will lay the foundation for future development of a novel therapy for children with CP.

## 1 Introduction

Cerebral palsy (CP) is a group of persistent central motor and postural development disorders and activity limitation syndromes caused by non-progressive brain damage in developing fetuses or infants, which is accompanied by sensory, perceptual, cognitive, communication, and behavioral disorders, as well as epilepsy and secondary muscle and bone problems. According to domestic and foreign reports, the prevalence of cerebral palsy is 1.4%~3.2%, and the prevalence of CP in children aged 1~6 years old in China is 2.46‰ (1, 2). The prevalence of CP in boys is higher than that in girls (2.25% vs. 1.59%), and higher in rural areas than in urban areas (2.75% vs. 1.90%).

There are significant disparities in the prevalence of CP across various geographical regions, and the incidence is observed to be increasing annually (3). The rehabilitation cycle for CP is prolonged and arduous, compounded by a shortage of rehabilitation educators (4, 5). According to the “Lancet” Global Burden of Diseases (GBD) study on rehabilitation published in 2020, China has over 460 million patients with rehabilitation needs, the highest of any country in the world, followed by India and the United States (6). According to data from the “China Health Statistics Yearbook” in 2022, the percentage of Chinese rehabilitation physicians (assistants) out of the total number of practicing (assistant) physicians is 1.3% in 2021 (7). Moreover, in many low-and middle-income areas, there exists a shortage of trained rehabilitation professionals, with fewer than 10 skilled practitioners per one million population (8). Rehabilitation equipment based on artificial intelligence (AI) technology is expected to solve the major bottleneck problem of insufficient rehabilitation teachers for CP. Therefore, we conducted a search across relevant research databases and information services on rehabilitation equipment for children with CP over the past 20 years. Subsequently, we reviewed the stimulation methods employed in conjunction with the equipment. The purpose is to provide a basis for the selection of stimulation methods for the development of rehabilitation equipment for children with CP. Through equipment research and development to solve the contradiction between the increasing demand for treatment of children with CP and the shortage of rehabilitation teachers. The development of AI will provide assistance for the development of intelligent decision support and intelligent treatment equipment.

## 2 Information and methods

### 2.1 Inclusion criteria

1. Research object: CP, there are no racial and gender restrictions.
2. Intervention measures: rehabilitation equipment for children with CP stimulation.
3. Rehabilitation equipment research and development stage: published research and development design or clinical application stage; the rehabilitation equipment entering the clinical application stage needed to have rigorous clinical trial design and verification of its safety or effectiveness.
4. Type of literature included in the study: original articles, Clinical Trials/CTs, randomized CTs/RCTs, non-RCTs, and conference papers.

### 2.2 Excluded criteria

1. Articles that do not involve children with CP.
2. Repeatedly published articles that are not related to the research topic.
3. Articles with missing data or the full text were not available.
4. Articles on rehabilitation equipment for children with CP where no stimulation method was described.
5. Articles other than Chinese and English were excluded.
6. Type of literature excluded in the study: reviews and commentaries.

### 2.3 Search strategy

The China National Knowledge Network Database (CNKI), the Wanfang database knowledge service platform, the Chongqing VIP information service, PubMed, Web of Science, Cochrane, ScienceDirect, Medline, Embase, and IEEE database were searched. The keywords were “cerebral palsy OR pediatric cerebral palsy,” “cerebral palsy rehabilitation equipment,” “cerebral palsy equipment,” “cerebral palsy intelligent tuina clothing,” “intelligent tuina suit for cerebral palsy,” “rehabilitation equipment,” and “stimulation mode.” The search strategy of the databases and information services and platforms is shown in Table 1.

**Table 1:** Search strategy for the database.

Query	Search term
#1	“Cerebral palsy OR Pediatric cerebral palsy”
#2	“Cerebral palsy rehabilitation equipment”
#3	“Cerebral palsy equipment”
#4	“Intelligent tuina suit for cerebral palsy”
#5	“Rehabilitation equipment”
#6	“Stimulation mode”
#7	#1 AND #2
#8	#1 AND #3
#9	#1 AND #4
#10	#1 AND #5
#11	#1 AND #2 AND #6
#12	#1 AND #3 AND #6
#13	#1 AND #4 AND #6
#14	#1 AND #5 AND #6

### 2.4 Literature screening method

**Figure 1:**
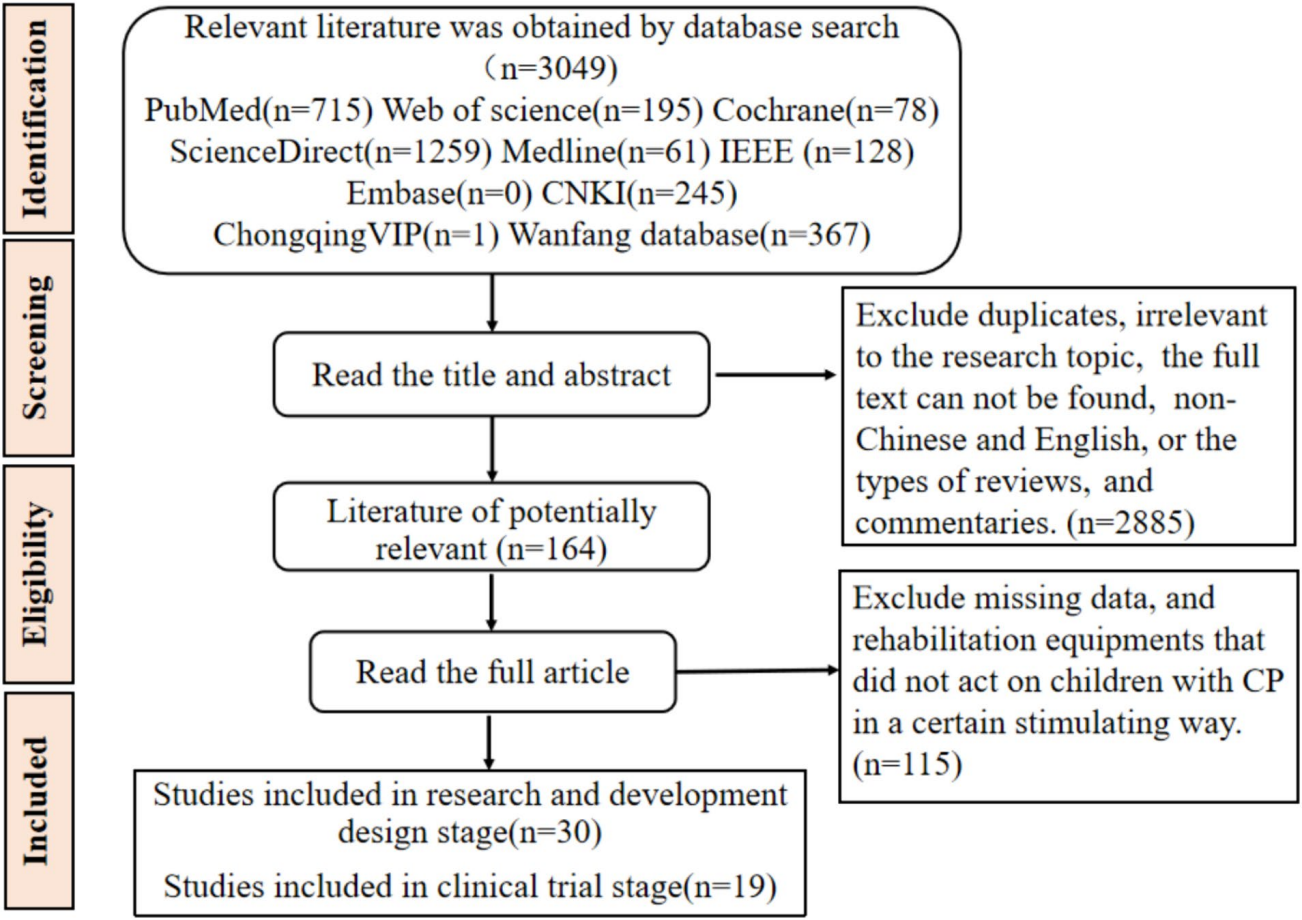
The flow chart of literature screening.

The search was for relevant articles published between 1 January 2003 to 1 December 2023. A total of 3,049 articles were retrieved. Through the application of inclusion and exclusion criteria and by reviewing the titles and abstracts, it was found that 2,885 articles were repetitive or unrelated to the research topic, or their full text was unavailable, or non-Chinese and English, or the types of reviews, and commentaries. We obtained 164 potentially relevant articles but after reading the full text, 115 articles did not meet the stated inclusion criteria and were excluded. The final number of relevant articles that adhered to the criteria and were included in this study was 49 articles (Figure 1).

In the literature screening, two researchers independently screened the relevant literature and extracted the data in strict accordance with the inclusion and exclusion criteria, and then cross-checked by two researchers to discuss the differences or ask third-party experts.

### 2.5 Methodological evaluation of clinical trials of rehabilitation equipment for children with CP

Two reviewers independently assessed the methodological quality of clinical trials of rehabilitation equipment for children with CP, using Physiotherapy Evidence Database (PEDro) Scale, PEDro scores were categorized as follows: <4, poor; 4~5, fair; 6~8, good; and 9~10, excellent (9).

### 2.6 Evaluation of evidence types in clinical trials of rehabilitation equipment for children with CP

We used the Oxford Center for Evidence-Based Medicine Levels of Evidence Working Group (OCEBM) to evaluate the evidence types in clinical trials of rehabilitation equipment for children with CP. It is divided into I (high-level evidence) to V (low-level evidence). Systematic reviews are usually classified as grade I clinical practice evidence, followed by randomized trials and blinded studies, which are classified as grade II. Grade III of cohort, follow-up and non-randomized studies; finally, the case series, case–control study, and mechanism-based reasoning are taken as the IV and V levels. Studies at levels I to IV may be downgraded due to research quality, inaccuracy, indirectness, inconsistency between studies, or small impact; if there is a significant impact, these studies may be escalated.

## 3 Results

### 3.1 Physical stimulation is the main stimulation method used with rehabilitation equipment for children with CP

**Figure 2:**
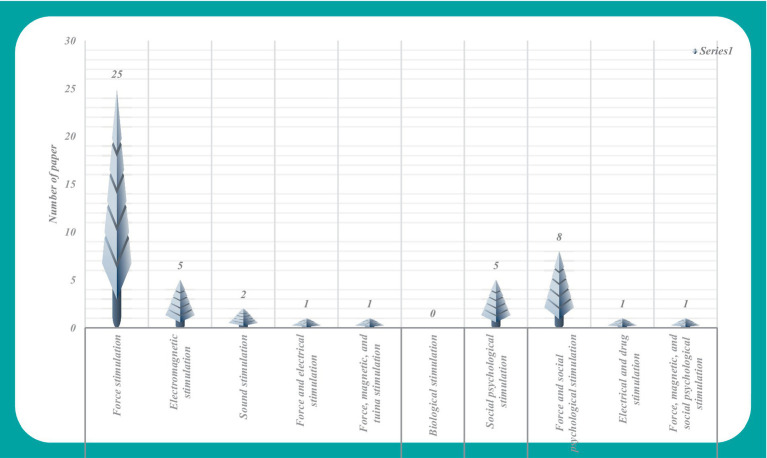
Literature analysis chart of stimulation methods used with rehabilitation equipment for children with CP.

The 49 articles that met the inclusion criteria were divided into four types according to the nature of the stimulation method used with the rehabilitation equipment. Physical stimulation included electricity, force, sound and light. Chemical stimulation included treatment with acids, alkalis and drugs. Biological stimuli included treatment with bacteria and viruses. The final type was psychosocial stimulation. According to the above classifications, 34 articles of physical stimulation were found, including 25 articles on force stimulation (10–34), 5 articles on electromagnetic stimulation (35–39), 2 articles on acoustic stimulation (40, 41), 1 article which combined force and electricity (42) and 1 article that combined force and magnetic tuina (43). There were no articles that employed biological stimulation, and 5 articles involved psychosocial stimulation (44–48). The other 10 articles reported a combination of two or more stimuli (49–56). One article used a combination of botulinum toxin stimulation with electrical and chemical stimulations (57), and another article used a combination of mechanical, magnetic, and psychosocial stimulations (58) (Figure 2).

In Figure 2, the analysis of research and development trends in rehabilitation equipment for children with CP reveals the prevalent use of force stimulation methods within the category of physical stimulation. The integration of force stimulation with psychosocial stimulation was the second most popular approach used in the relevant literature. However, it is noteworthy that the application of alternative stimulation methods appears comparatively limited within the current landscape of research and development in this field.

**Figure 3:**
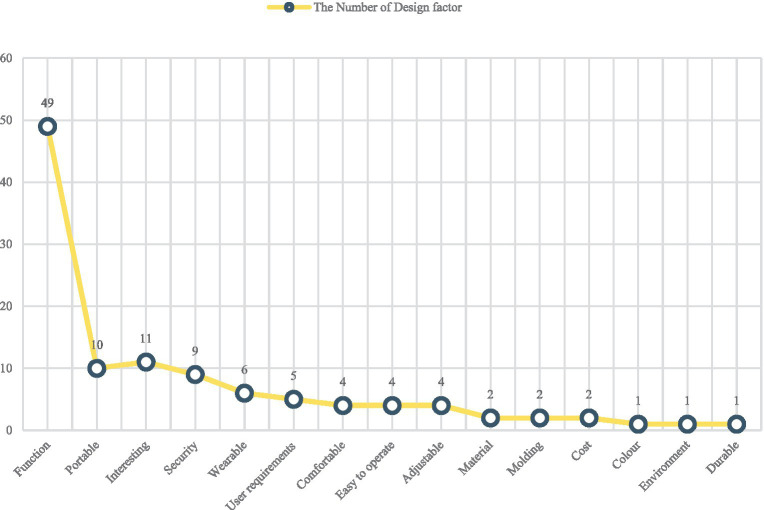
The chart of design factor.

Figure 3 shows the design factors described in the 49 relevant articles. These design factors included functionality of the equipment, interest, portability, safety/security, wearability, user requirements, comfort, ease of operation, adjustability, materials, modeling, cost, color, and durability. The analysis of the design factors, revealed the design factor that was most prevalent was the function of the equipment. The other design factors were related to the experience of the children with CP using the equipment and the safety of the equipment, which provided a reference for the proposed future research and development of rehabilitation equipment for children with CP.

The data from Figures 2, 3 show that articles involving force stimulation constitute 51.02% of the research corpus to date. This may be attributed to the perceived advantages of force stimulation in terms of simplicity and ease of implementation. Noteworthy factors considered in device development include design elements such as interest, portability, safety, and wearability, which underscore the multifaceted approach to advancing this field of research.

### 3.2 Analysis of force stimulation

Force stimulation emerges as a prevalent modality within the category of physical stimulation because it offers versatility across various applications. The literature review revealed specific force stimulation modes, which included auxiliary fixation force, weight-bearing force, and the utilization of exoskeleton robots or rehabilitation robots. An exhaustive analysis of force stimulation methods is conducted within the framework of these four force stimulation modes and is presented in Tables 2, 3.

**Table 2:** Analysis table of force stimulation mode of rehabilitation equipment for pediatric CP.

Stimulation mode	Stimulation methods	Improved functionality	References
Auxiliary fixed force	Adjusting the sitting position.	Improve the ability to sit or adjust abnormal sitting posture.	(10)
	Correct upper and lower limb posture and promote lower limb movement through assistive devices and suspension devices.	Correct the abnormal posture of lower limbs in patients with CP, and gradually establish normal standing and walking function.	(11)
	Auxiliary fixed spine.	Seating systems exert influence body posture and postural control, However, the best seating choice for each child should be selected individually.	(12)
Weight-bearing force	Adjust the load by putting sand or water into the storage chamber.	Improve walking and balance ability.	(13)
Auxiliary device passive force	Training is carried out with a rack, gear, bearing, and hydraulic cylinder driving device.	Used for holistic rehabilitation training and balance ability of the hand, leg, and foot in patients with CP.	(14)
The power supply drives the leg, twist waist, and top waist device.	Used for upper and lower limb movement of children with CP.	(15)
Through a cable, spindle, pulley system, and brace with mounted load cells. The DC motor can provide up to 5 Nm of torque continuously about the wrist.	A lightweight low clearance wrist orthosis for use in children with CP that actuates pronation/supination and flexion/extension of the wrist.	(16)
The device could provide adjustable resistances, which could be scaled based on a subject’s ability.	PaRRo is a feasible approach to provide functional resistance training to the muscles along the upper extremity.	(17)
A hybrid rehabilitation tricycle includes three major subsystems: mechanical subsystem, electronic subsystem, and software subsystem.	Hand and leg cycle rehabilitation.	(18)
The lower body positive pressure system (AlterG) consists of a treadmill that is enclosed in an inflatable bag. The amount of body weight support depends on the air pressure in the bag, which establishes a vertical force (opposite direction of gravity) to the body.	AlterG training can improve the lower limb performance and locomotion in children with CP.	(19)
The WAKE-up mechanical system, control architecture and feature extraction are described. Two test benches were used to mechanically characterize the device. The full system showed a maximum value of hysteresis equal to 8.8% and a maximum torque of 5.6 N m/rad.	WAKE-up assisted patients in recovering the physiological gait patterns.	(20)
The AlterG system consists of six main parts, including a treadmill, a transparent and inflatable chamber, a force plate to measure patient’s weight, two compressors, a controller, and a pair of neoprene shorts.	AlterG training appears to be a robust therapeutic intervention to reduce neuromuscular abnormalities and manage spasticity.	(21)
Using rhythmic movement mode, air pressure automatically drives the affected metacarpophalangeal joint and interphalangeal joint to repeat passive grasping and releasing activities.	Application of pneumatic hand rehabilitation equipment in comprehensive rehabilitation training can effectively promote the recovery of hand function for children with spastic hemiplegic cerebral palsy.	(22)
Upper limb exoskeleton	Developed a Bilateral ADL Exercise Robot, BiADLER aimed at training children with CP in reach to grasp coordination on ADLs.	The design is tuned to support the retraining of wrist motions that are targeted by the wrist tendon surgery.	(23)
	PEXO provides both wrist motion capability of 60° in flexion and extension and wrist stabilization at the same time, and a compliant wrist mechanism extending the existing leaf spring finger mechanism of the device.	The adjustability in the wrist enables a larger variety of grasping gestures.	(24)
	Actuator-and-control module, a Bowden cable and pulleys that are controlled using a real-time control algorithm and embedded sensors. An average of 0.26 Nm·kg-1 of plantar-flexor assistance.	Improvement in the metabolic cost of transport during walking with untethered exoskeleton assistance compared to how participants walked normally.	(25)	Lower extremity exoskeleton
	The powered exoskeleton consists of a motor and transmission assembly that is mounted laterally to custom molded thermoplastic foot, shank, and thigh orthotics. It provided an assistive torque about the knee joint. The exoskeleton had a passive, adjustable ankle joint which was set to free rotation.	Indicates the importance of properly tuned robotic control strategies for gait rehabilitation.	(26)	
	The exoskeleton, based on the architecture of a knee-ankle-foot orthosis, is lightweight (3.2 kg) and modular. On board sensors enable knee extension assistance to be provided during distinct phases of the gait cycle.	The potential for a powered exoskeleton to reduce excessive knee flexion during crouch gait from CP.	(27)	
	Brushless DC motors and harmonic drive gears were chosen for their efficiency, and an EtherCAT communication protocol was employed to enable high-frequency, responsive operation. The exoskeleton, managed by a high-performance embedded controller, is equipped with distinctive mechanical, sensory, and control features, offering personalized support considering the wide range of muscle tone and spasticity in children with CP.	The controlled Single-Leg Exoskeleton (SLE) can enhance the walking functionality of children with CP, enabling them to accurately follow predefined target trajectories.	(28)	
	Utilized an untethered, battery-powered ankle exoskeleton, this device consisted of an assembly worn at the waist and bilateral ankle assemblies. The motor and control assembly actuated the ankle pulleys via Bowden cables, allowing the device to resist ankle plantar flexion. The research team was able to wirelessly control the level of resistance delivered by the device via Bluetooth and a custom MATLAB graphical user interface.	The findings underscored the importance of monitoring how users change their gait kinematics when walking with the resistive device, with a specific emphasis on stance-phase lower limb extension.	(29)	
	The device was composed of a control system, coding actuator, and exoskeleton support. Under the control of the system, the coding actuator drove the exoskeleton support to perform passive rehabilitation movements in the corresponding mode. The device enables the acetabulum and femoral head of the child to perform hip joint rehabilitation exercises at 5~16/s in the range of 50°~110°.	The passive lower limb rehabilitation training equipment developed in this paper can be used for rehabilitation training after Spastic cerebral palsy hip dislocation in children, intervened in the process of hip dysplasia, reduced the risk of hip dislocation prevention, and improved the children’s lower limb movement ability.	(30)	
	The Lokomat is a bilaterally driven exoskeleton, combined with a BWS system and a treadmill. It is actuated by linear drives to provide “guidance” to the legs, and follows a reference pattern based on the kinematics of walkers.	The amplitude of activity was lower in the Lokomat than on the treadmill. During Lokomat walking, providing guidance and BWS resulted in slightly lower amplitudes whereas increased speed was associated with higher amplitudes.	(31)	
	Ankle exoskeleton is composed of an actuation and control assembly, force sensors, and an onboard microcontroller. It provided proportional resistance to ankle plantar flexion.	Ankle exoskeleton resistance therapy shows promise for rapidly improving neuromuscular control for children with CP.	(32)	
Upper limb rehabilitation robot	REAPlan is fitted with force and position sensors (acquisition frequency = 100 Hz), allowing for control of the lateral and longitudinal interaction forces between the patient and the robot. The patient has to perform the movement along a reference trajectory.	Robot-assisted therapy (RAT) improved upper limb kinematics and manual dexterity.	(33)
Lower limb rehabilitation robot	This allows children of any ability to move on the ground using wearable robot legs and connects external power to the walker, providing safety and stability.	Regular use of the Trexo Home robotic gait trainer has positive outcomes on frequency and quality of bowel movements, and may improve head control, and knee flexor spasticity.	(34)

**Table 3:** Analysis table of force combined with other forms of stimulation of rehabilitation equipment for pediatric CP.

Stimulation mode	Stimulation methods	Improved functionality	References
Force/Electricity Stimulation	Stimulate acupuncture points with a combination of tuina claws and electrical stimulation.	Promote the rehabilitation of CP.	(42)
Force/Magnetic /tuina	Cylinder drive, magnet body tuina, and force-assisted walking.	Promote the recovery of walking function in children with CP.	(43)
Force and Social Psychological Stimulation	Force/game stimulation/psychosocial stimulation.	Improve gross motor and cognitive ability of children with CP.	(49)
	The lower limb rehabilitation robot is driven by a motor for training.	Improve gross motor and balance function in children with CP.	(50)
	Force stimulation combines the unique advantages of gamification, enhances the fun and interactivity of training, and transforms training from passive to active.	Improve hand function in children with mild CP.	(51)
	Force and Social Psychological Stimulation.	Improve rehabilitation of ankle joint in children with CP.	(52)
	Motor-driven lower limb rehabilitation robot combined with virtual reality game settings for training.	Improve the walking ability of children with CP.	(53)
	The MaKey uses high resistance switching to detect when a user makes a connection.	Motivating and increasing such individuals’ physical activity of CP.	(54)
	The robot had two major modules for intervention options, one was to provide strenuous and safe stretching controlled by an intelligent-control algorithm and the other trained voluntary movement control of the patients. In passive stretching, the stretching velocity was inversely proportional to the joint resistance torque and slowed down with increasing resistance.	The positive results of this study were improvements in motor control and motor function.	(55)
	The device includes a motor and transmission system, mounted to a knee-ankle-foot orthosis. The exoskeleton contains on board sensors to measure knee angle, knee torque, and foot contact which allow for feedback control of extension assistance. It was combined with a video game.	The exoskeleton significantly increased knee extension.	(56)
Force/magnetic/social psychological stimulation	The wrist dorsiflexion training was driven by the combination of battery, magnetic beads, metal probes, and music.	Improves the range of dorsiflexion and fine motor ability of children with CP.	(58)

#### 3.2.1 Analysis of force stimulation mode

Table 2 shows that the stimulation method of force has evolved from the initial fixed force and weight-bearing force of the auxiliary device to the passive force of the auxiliary device, and finally to the upper and lower limb exoskeleton robot/lower limb rehabilitation robot. Force stimulation has been quantified from no quantification to further quantification within the range of joint functional activity. Different amounts of stimulation have different focuses on the improvement of function. The auxiliary fixation force is mainly used to correct the posture and gradually establish the walking function whereas the weight-bearing force mainly improves walking and balance function. The power of the auxiliary device is quantified in the amount of force stimulation, and the function is gradually improved. This is mainly used to improve the motor function of the upper and lower limbs, followed by the improvement of muscle strength training, blood circulation, and balance coordination function. The upper and lower extremity exoskeletons and rehabilitation robots are more abundant and perfect in the realization of functions. Targeted training is carried out for the flexion and extension of a specific joint such as the wrist, knee, and ankle in the upper and lower limbs. Even some devices can also increase the repeatability and interest of children with CP in limb function training based on certain task settings.

Table 3 reveals that the amalgamation of force-based stimulation with electrical, magnetic, tuina, and psychosocial stimulation modes yields a synergistic effect. This integrated approach proves beneficial in enhancing cognition, movement, balance, gait, and overall physical function among children with CP, which fosters a heightened interest in their training endeavors. The combination of stimulation methods aligns with the distinctive characteristics of individual pediatric patients, and addresses pertinent clinical requirements. However, a noteworthy limitation lies in the insufficient consideration of the equipment’s applicability scope and adjustability. Simultaneously, it is worth noting that the force stimulation of simulated tuina manipulation is utilized in the fusion of multiple stimulation methods. The effect of tuina manipulation is based on the fundamental theory of traditional Chinese medicine (TCM), which stimulates certain parts or acupoints of the human body to achieve the effect of preventing and treating diseases. Nevertheless, how to objectively quantify the amount of stimulation and repeatability needs to be solved.

#### 3.2.2 Analysis of the research and development phase

The research and development (R&D) of equipment is generally divided into four stages according to its development stage. The first stage is prototype development where the main consideration is how many devices have been certified by the Food and Drug Administration. The second stage is the feasibility study, where the number of trials is small and usually with a prototype device to assess its safety and clinical feasibility. The third stage is the clinical trial, which determines the effectiveness of the treatment. The final stage is the commercialization stage, which can be popularized and applied (59). In this article, there are 19 articles involving devices entering the clinical trial stage, of which 17 are force stimulation-based devices. Table 4 shows that the sample size of the test is smaller in the 17 force stimulation-based literatures. The intervention measures were mainly based on original equipment, and the outcome indicators were mainly gross motor function measures (GMFM-88), Fine motor function measure (FMFM), Modified Ashworth scale (MAS), Modified Tardieu Scale (MTS), etc. Data from the clinical trial of the equipment, verified the effectiveness of the equipment, which laid a foundation for its popularization and wide application. However, during each R&D stage there is a better understanding of the existing equipment including its limitations and frequently there needs to be modifications and improvements. Thus the equipment usually undergoes a long process from R&D to listing.

**Table 4:** Clinical research analysis table of force stimulation-based rehabilitation equipment.

Included literature	Age	Sample size		Intervention measures		Course of treatment	Outcome measures
		T	C	T	C		
Zhou YP 2018 (14)	3.5 ± 1.5 years	108	108	A self-developed rehabilitation training device combined with group C treatment.	Routine symptomatic treatment and Monosialotetrahexosy1 ganglioside combined with brain protein hydrolysate.	1 time/day, 40 min/time, 12 months.	GMFM-88, the comprehensive evaluation of Wee-Function Independence Measure (WeeFIM) Scale.
Lotfian M 2017 (19)	9~13 years	4	0	(AlterG) training.	/	40 min/time, 3 times/week, 8 weeks.	The kinematics and kinetics were evaluated using an isokinetic dynamometer.
Patane F 2017 (20)	5~13 years	3	4	HC group: Normal Walking without the exoskeleton, walking with exoskeleton with the motor switched off/motor on. Each condition was repeated.	TDC group: walking without the exoskeleton repetitioned five times.	45 min/time, five times	16 retro-reflective markers, NW-MON.
Noroozi S 2020 (21)	4~14 years	9	9	AlterG	Conventional occupational therapy.	40 min/time, 3 times/week, 8 weeks.	Reflex stiffness gain (GR) and intrinsic stiffness gain (K).
Gu QY 2023 (22)	3~5y	29	29	Pneumatic hand rehabilitation equipment training combined with group C treatment.	Comprehensive rehabilitation training	20 min/time, 1 time/day, 5 days/week, 6 months.	Developmental Motor Scales-Fine Motor (PDMS-FM), FMFM, and Wee-FIM.
Lerner ZF 2018 (25)	5~30 years	5	0	Assisted exoskeleton walking	/	Evaluation twice and assessment visits, 4~10 exoskeleton training visits. 25 min/day, 10 training, rest 5~10 min, 130 min.	A shod (shoes only) trial, and walking trial analyzed in this study took place on an in-ground treadmill (Bertec) and lasted for 5 min.
Lerner ZF 2017 (26)	5~19 years	4	0	Walked with assistance from a novel robotic exoskeleton on an instrument ed. treadmill.	/	Six visits.	Infrared motion capture camera-instrumented treadmilland Electromyography (EMG)
Conner BC 2022 (29)	12~18 years	8	0	Resistive ankle exoskeleton	/	Visits 1~6 took place over a two-week period, with 24~72 h between visits. Visit 7 was completed 2 weeks.	Surface electromyography (sEMG) signal, smoothed via an 80 ms moving average filter.
Van Kammen, K 2020 (31)	6~16 years	10	0	Walked on a treadmill and in the Lokomat guidance	/	9 trials (a trial on the Lokomat treadmill without the exoskeleton attached, eight trials in the Lokomat exoskeleton)	Electromyography and detection of gait events
Conner, BC 2021 (32)	12~17 years	5	0	Exoskeleton ankle resistance therapy (exo-therapy)	/	20 min/session, 10 times, 4 weeks.	Surface electromyography, three-dimensional kinematics, and metabolic data
Gilliaux, M 2015 (33)	﹤18 years	8	8	Robot-assisted therapy (RAT) and CT sessions	Conventional therapy (CT) session	C, 5 CT sessions/week, T, 2 RAT and 3 CT sessions /week, 45 times/session, 8 weeks	International Classification of Functioning (ICF) Assessment and 3 questionnaires
Deng HY 2019 (49)	﹤6y	6	7	Wearable device games training Combined with group C training.	Conventional rehabilitation training.	45 min/time, 2 times/week, 8 weeks.	GMFM-88 and Wechsler Intelligence Scale for Children Fourth Edition (WPPSI-IV).
Cheng JH 2021 (50)	4~9 years	60	60	Conventional rehabilitation treatment and lower limb rehabilitation robot training.	Conventional rehabilitation treatment.	30 min/time, 1 time/day, 6 times/week, 12 weeks.	GMFM-88, Berg balance scale (BBS)
Wang RL 2018 (52)	4~13 years	6		A robot-assisted ankle-foot rehabilitation system.	/	10 cycles/group, 5 groups/person. Each group interval of 3 min.	MTS and joint biomechanical properties (ankle plantar flexion resistance torque under different ankledorsiflexi on angles).
Fu WS 2020 (53)	6~11 years	15,15,15	15	Virtual reality walking robots with group C training.	Conventional rehabilitation training.	The two group received routine rehabilitation training for 30 min, Group C increased walking for 20 min, while group T combined with Lokomat walking for 20 min. 5 times/week, 12 weeks.	GMFM-88, six-minute walking test (6-MWT), Berg balance scale, MTS, gastrocnemius surface EMG, and the bone density of the calcaneus of the affected lower limb.
Bulea TC 2017 (56)	5~19 years	6	0	The combination of an assistive exoskeleton and an exercise video game training.	/	knee extension and knee flexion exercise lasting a total of 40 s for each trial of game play. Six total visits lasting 2~4 h each.	kinematic, EMG, and EEG
Tan LS 2017 (58)	1~3 years	28	27	Equipped with self-made music cartoon wrist rehabilitation training device, and group C training.	Conventional rehabilitation training.	1 time/day, 10 min/time, 6 days/week, 1 month.	Wrist dorsiflexion range of motion (ROM) and FMFM.

#### 3.2.3 Evaluation of clinical trial methodology and evidence type of force stimulation equipment

In order to further evaluate the entry of 17 force-stimulated devices into clinical trials, the clinical trial methodology was evaluated using the PEDro Scale (Table 5). OCEBM was used to analyze the type of evidence (Table 6).

**Table 5:** PEDro scale total scores of force-stimulated devices clinical trials.

Article	PEDro Scale										
	Random allocation	Concealed allocation	Baseline comparability	Blind subjects	Blind therapists	Blind assessor	Adequate follow up	Intention-to-treat-analysis	Between-group comparisons	Point estimates and variability	Total score
Zhou YP 2018	YES	NO	YES	NO	NO	NO	NO	NO	YES	NO	3 (Poor)
Lotfian M 2017	NO	NO	YES	NO	NO	NO	YES	NO	YES	YES	4 (Fair)
Patane F 2017	NO	NO	YES	NO	NO	NO	NO	NO	YES	YES	3 (Poor)
Noroozi S 2020	NO	NO	YES	NO	NO	NO	YES	YES	YES	YES	5 (Fair)
Gu QY 2023	YES	NO	YES	NO	NO	NO	NO	YES	YES	YES	5 (Fair)
Lerner ZF 2018	NO	NO	YES	NO	NO	NO	NO	YES	YES	YES	4 (Fair)
Lerner ZF 2017	NO	NO	YES	NO	NO	NO	NO	YES	YES	YES	4 (Fair)
Conner BC 2022	NO	NO	YES	NO	NO	NO	NO	YES	YES	YES	4 (Fair)
Van Kammen, K 2020	NO	NO	YES	NO	NO	NO	NO	YES	YES	YES	4 (Fair)
Conner, BC 2021	NO	NO	YES	NO	NO	NO	NO	YES	YES	YES	4 (Fair)
Gilliaux, M 2015	YES	NO	YES	NO	NO	YES	NO	YES	YES	YES	6 (Good)
Deng HY 2019	YES	NO	YES	NO	NO	NO	NO	YES	YES	YES	5 (Fair)
Cheng JH 2021	YES	NO	YES	NO	NO	NO	NO	YES	YES	YES	5 (Fair)
Wang RL 2018	NO	NO	YES	NO	NO	NO	NO	NO	YES	YES	3 (Poor)
Fu WS 2020	YES	NO	YES	NO	NO	NO	NO	YES	YES	YES	5 (Fair)
Bulea TC 2017	NO	NO	YES	NO	NO	NO	NO	NO	YES	YES	3 (Poor)
Tan LS 2017	YES	NO	YES	NO	NO	NO	NO	NO	YES	YES	4 (Fair)

Include criteria-YES; exclude criteria-NO.

**Table 6:** Levels of evidence used to justify force-stimulated devices clinical trials.

Article	OCEBM levels of evidence				
	Leve I	Level II	Level III	Level IV	Level V
Zhou YP 2018		II			
Lotfian M 2017				IV	
Patane F 2017			III		
Noroozi S 2020			III		
Gu QY 2023		II			
Lerner ZF 2018				IV	
Lerner ZF 2017				IV	
Conner BC 2022			III		
Van Kammen, K 2020				IV	
Conner, BC 2021				IV	
Gilliaux, M 2015		II			
Deng HY 2019		II			
Cheng JH 2021		II			
Wang RL 2018				IV	
Fu WS 2020		II			
Bulea TC 2017				IV	
Tan LS 2017		II			

Based on the OCEBM Levels of Evidence. Level I, Systematic review of randomized trials, systematic review of nested case–control studies; Level II, Individual randomized trial and blinded studies or (exceptionally) observational study with dramatic effect; Level III, cohort, follow-up and non-randomized studies; Level IV, Case-series, case–control, or historically controlled studies; Level V, Mechanism-based reasoning.

Table 5 shows that the methodological level of clinical trials of force stimulation equipment is mainly 4–5 (Fair), and the level of 6–8 (Good) is limited. Table 6 suggests that there are 7 articles of Level 2 and Level 4 in the evidence type of force stimulation clinical trials, and 3 articles of Level 3. Tables 5, 6 show that high-level evidence types are not necessarily high-quality methodologies. Therefore, in order to make the results more reliable in the clinical trial design of pediatric cerebral palsy equipment, the methodology needs to be further improved.

## 4 Discussion

The World Health Organization released information that 50% of rehabilitation needs have not been met, and there are inadequate numbers of rehabilitation physicians for children with CP particularly in low-and middle-income countries. With the development of AI, the R&D of rehabilitation equipment combined with medicine and engineering has shown a vigorous trend of development. Children with CP have a long rehabilitation cycle because of their life-long motor disability, as well as a wide variety of associated impairments.

The R&D of rehabilitation equipment for adjuvant treatment of children with CP has emerged as a prominent focus of research and is increasingly capturing public attention. The stability, durability, and reproducibility of AI technology (60) are highly likely hold significant importance for the treatment of CP. Equipment development can be tailored for children with regard to the mode of stimulation and the amount of stimulation, which can be quantified and controlled. This paper analyzes the stimulation methods of rehabilitation equipment for children with CP, to provide a reference for the R&D of rehabilitation equipment for children with CP.

Upon assessing the relevant articles identified in our literature search, it is evident that the stimulation mode of the equipment primarily revolves around physical stimulation. Specifically, force stimulation is widely employed. This approach offers the advantages of straightforward operation, a broad application range, and the absence of toxic side effects associated with physical factor stimulation (61). Force stimulation is easily achievable, controllable, and devices employing force stimulation, as featured in 17 of the relevant articles, have undergone clinical trials to assess their efficacy. The essential prerequisites for these trials include the design of sample size, the test plan, treatment course, and outcome index, all of which yield reliable data crucial for driving the subsequent development of equipment. However, in the follow-up studies, the sample size, methodological quality and evidence type of equipment clinical trials need to be strengthened. The integration of force stimulation with other stimulation methods can harness the advantages of various approaches, particularly the combination of force and social-psychological stimulation, aligning more closely with the modern medical model’s development. Some articles include electromagnetic stimulation, psychosocial stimulation, and acoustic stimulation. Electromagnetic stimulation offers the advantage of simplicity and ease of implementation. Especially for stroke treatment is widely used, such as tDCS can be easily applied simultaneously with other therapies (62). It has good tolerance, safety, portability, and suitable for different levels of patients (63), but the stimulation threshold for children with CP requires further refinement. Non-invasive electromagnetic stimulation presents an appealing option for treating children with CP, yet the safety requirements necessitate further strengthening. Psychosocial stimulation is utilized independently, and its functionality is relatively straightforward. Articles employing acoustic stimulation predominantly utilize ultrasonic stimulation. The application of acoustic stimulation in cerebral palsy rehabilitation equipment remains relatively limited and is still in its infancy. Acoustic stimulation offers certain advantages in regulating neurological function, Studies have confirmed that noninvasive ultrasound deep brain stimulation (UDBS) enables modulation of the subthalamic nucleus (STN) or the globus pallidus (GP) neural activity and leads to neuroprotection in PD mice, potentially serving as a noninvasive strategy for the clinical treatment of PD (64). Noninvasive modulation of neuronal activity is an important translational application of focused ultrasound (FUS). it elicited action potentials with millisecond latencies compared with electrical stimulation, suggesting ion channel-mediated mechanisms (65). However, its application for children with CP requires clarification. Furthermore, stimulation parameters as well as safety thresholds necessitate further definition with this method.

The search described in this paper possesses two limitations. Firstly, a relatively small number of potentially relevant articles were excluded due to the unavailability of full texts. Secondly, variations in professional perspectives may result in disparities in article inclusion, data extraction, and analysis.

In conclusion, research on the stimulation mode of rehabilitation equipment for children with CP is mainly based on the force stimulation in physical stimulation. The force used is mainly based on external forces such as fixation, correction, and active or passive movement. The R&D of equipment with more choice of force stimulation conforms to the advantages and characteristics of force. At present, the force stimulation method of simulating pediatric tuina manipulation on children with CP is less used. With the development and integration of AI, the research on the stimulation method of rehabilitation equipment for children with CP will focus on the force of simulating tuina manipulation, and the combination of AI and personalization will be the trend for future equipment development. The application of this stimulation method can not only objectively quantify the stimulation amount of tuina manipulation, but also make treatment by tuina manipulation uniform and reproducible, and thereby benefit from this unique form of Chinese traditional medicine. Thus, AI is likely to go a long way in solving the bottleneck problem of insufficient numbers rehabilitation teachers.

At present, through the combination of TCM and AI, some scholars have objectively quantified the four diagnostic methods of TCM (observation, auscultation, inquiry and palpation), developed an expert decision support system for children with CP, realized intelligent diagnosis, and solved the objectification of the four diagnostic methods of TCM for children with CP, the standardization of syndrome differentiation and the intelligentization of rehabilitation decision-making (66). The key to intelligent “treatment” is the key technologies such as acupoint selection (67), manipulation selection, intelligent recognition and automatic adjustment of acupoints in TCM. Finally, an intelligent tuina device for the diagnosis and treatment of CP in children is formed, which lays a foundation for the construction of a three-level linkage intelligent rehabilitation network for cerebral palsy such as hospital-grassroots community-family.

## Author contributions

CG: Writing – review & editing, Writing – original draft, Formal analysis, Data curation, Conceptualization. YoC: Writing – review & editing, Writing – original draft, Formal analysis, Data curation, Conceptualization. BX: Writing – original draft, Writing – review & editing. SC: Writing – original draft, Methodology. CZ: Writing – original draft, Methodology. YiC: Writing – original draft, Methodology. ES: Writing – original draft, Methodology. PZ: Supervision, Writing – original draft. XT: Writing – original draft, Supervision.
